# Improving Time-to-Treatment for Advanced Non-Small Cell Lung Cancer Patients through Faster Single Gene EGFR Testing Using the Idylla™ EGFR Testing Platform

**DOI:** 10.3390/curroncol29100624

**Published:** 2022-10-18

**Authors:** Norbert Banyi, Deepu Alex, Curtis Hughesman, Kelly McNeil, Diana N. Ionescu, Carmen Ma, Stephen Yip, Barbara Melosky

**Affiliations:** 1 Faculty of Medicine, University of British Columbia, Vancouver, BC V6T 1Z3, Canada; 2 Department of Pathology, BC Cancer, Vancouver, BC V5Z 4E6, Canada; 3 Department of Pathology and Laboratory Medicine, University of British Columbia, Vancouver, BC V6T 1Z7, Canada; 4 Cancer Genetics and Genomics Laboratory, BC Cancer, Vancouver, BC V5Z 4E6, Canada; 5 Medical Oncology, BC Cancer, Vancouver, BC V5Z 4E6, Canada

**Keywords:** EGFR testing, time-to-treatment, non-small cell lung cancer, genetic testing, lung molecular biomarkers

## Abstract

Introduction: Patients with advanced-stage non-small cell lung cancer (NSCLC) may benefit from a short time-to-treatment (TTT). Predictive biomarker testing is performed prior to treatment, as recommended by various international expert consensus bodies. Genetic testing is more time-intensive than immunohistochemistry (IHC) and commonly contributes to prolonged TTT. For epidermal growth factor receptor-positive patients (EGFR+), further genetic testing may not be required due to the mutual exclusivity of actionable mutations. Methods: The trial cohort (N = 238) received both BC Cancer NGS panel (Oncopanel) and Idylla EGFR testing. Data were also collected for a control cohort (N = 220) that received Oncopanel testing. For each patient, the time that the lab received the sample, the time taken to report the NGS and Idylla tests, the time of first treatment, and the final treatment regimen were recorded. Results: A concordance frequency of 98.7% (232/235) was observed between the Idylla and NGS panel. The lab turnaround time (TAT) was faster for the Idylla test by an average of 12.4 days (N = 235, *p* < 0.01). Overall, the average TTT in the trial cohort (N = 114) was 10.1 days faster (*p* < 0.05) than the control (N = 114), leading to a 25% reduction in TTT. For patients treated based on EGFR positivity, the mean TTT was 16.8 days faster (*p* < 0.05) in the trial cohort (N = 33) than the control cohort (N = 28), leading to a 48% reduction in TTT. Conclusion: Using the Idylla EGFR test as part of the molecular testing repertoire in advanced-stage NSCLC patients could significantly reduce TTT.

## 1. Introduction

Patients with advanced-stage non-small cell lung cancer (NSCLC) often suffer from rapid disease progression and may benefit from a shorter time-to-treatment (TTT). The treatment for advanced-stage NSCLC has progressed in the past decade, relying on a personalized approach to deliver optimal treatment. Predictive biomarker testing is performed prior to treatment as recommended by various international expert consensus bodies, including guidelines published by the College of American Pathologists (CAP), the International Association for the Study of Lung Cancer (IASLC) and the Association for Molecular Pathology (AMP) [1], American Society of Clinical Oncology and Ontario Health (Cancer Care Ontario) NSCLC Expert Panel [2], and the National Comprehensive Cancer Network (NCCN) [3]. Recommended molecular testing includes screening for key actionable mutations (*EGFR*, *ALK*, *ROS1*, *BRAF*, *MET*, *NTRK*, *KRAS*, etc.) and PD-L1 [1]. Traditionally at BC Cancer, actionable mutations in NSCLC were tested using a 41-gene next generation sequencing (NGS) panel called Oncopanel, for which the mean lab turnaround time (TAT) was previously estimated to be 14 days [4]. This is a hybrid-capture based DNA panel that detects mutations in *EGFR*, *KRAS* and *MET,* but requires at least 200 nanograms of DNA with a minimum tumor content of 20%. Biomarkers for actionable fusion events such as ALK and ROS1 are tested by immunohistochemistry (IHC) and fluorescent in situ hybridization (FISH), which are evaluated within one week of request, or earlier. As such, the bottleneck for molecular testing is the genetic testing component. One approach to improving time-to-treatment (TTT) would be through decreasing lab TAT for genetic testing. 

Mutations in the *EGFR* gene are commonly observed in advanced-stage NSCLC patients who have lung AC with an overall incidence of about 30% [5], but this rate can vary based on ethnicity [6,7]. In the Canadian NSCLC patient population, where EGFR testing only in patients with potential or diagnosed advanced non-squamous NSCLC has been the standard of care, the estimated incidence of *EGFR* mutations is 15% [8,9]. Tyrosine kinase inhibitors (TKI) have shown to be effective for use against EGFR-driven NSCLC used as the first-line treatment in patients positive for various *EGFR* mutations [10]. When targetable treatment is not appropriate, immunotherapy is recommended for patients that have PD-L1 expression in more than 50% of tumor cells [11]. Prior to treatment based on PD-L1 status, it is important to know if the patient has an *EGFR* mutation. Immunotherapy in patients with EGFR-driven NSCLC is not effective and switching from immunotherapy to EGFR-TKI treatment can result in severe complications or death [12,13]. Reducing the TAT to determine *EGFR* status by molecular testing may also reduce the TTT with immunotherapy in appropriate patients.

*EGFR* mutations are functionally mutually exclusive with other single oncogenic drivers, enabling oncologists to act on positive *EGFR* results without needing test results for other mutations [14,15,16,17,18]. As such, ultra-rapid *EGFR* testing may improve TTT by decreasing lab TAT for genetic testing. In cases where patients are PD-L1-high (PD-L1 ≥ 50%), a rapid *EGFR* result could allow oncologists to correctly begin immunotherapy while comprehensive testing is still under way. Previous studies have demonstrated the high concordance of the Idylla testing platform with established testing methods, which ranges from 94% to 100% [19,20,21,22,23,24,25,26]. While it has been shown that the Idylla EGFR test reduces the time to EGFR mutation result in NSCLC patients, there is yet to be a comparison of TTT between patients who receive Idylla EGFR testing and those who receive only NGS panel testing [27].

The single gene Idylla assay costs about CAD 250 per test, whereas the 41-gene NGS assay is slightly more than CAD 1000 per test. Although the Idylla EGFR assay is relatively expensive for information on a single gene, it potentially reduces treatment costs when the right therapy can be instituted faster and in the right order, whether that be targeted therapy or immunotherapy. Moreover, if implemented in a stepwise manner such that comprehensive NGS panel testing is only initiated after a negative EGFR test, it can result in overall time and cost savings for patients positive for EGFR mutations. Finally, the Idylla assay can handle specimens with lower tumor content, which can potentially prevent the need for a re-biopsy.

To evaluate the feasibility of incorporating the Idylla *EGFR* test into a molecular testing workflow for improving TTT, this study looked at three objectives. First, to validate the results of the Idylla *EGFR* test and compare them with the Oncopanel NGS test. Second, to compare time taken from a sample received at the testing laboratory to report generation (lab TAT) using Idylla or Oncopanel. Third, to analyze the difference between the TTT in patients who received Idylla *EGFR* testing and a control group of patients who only received Oncopanel testing. 

## 2. Materials and Methods

### 2.1. Setting and Participants

Data were collected for a retrospective control cohort (N = 220) that only received an Oncopanel test and a prospective trial cohort (N = 238) that received both an Oncopanel and Idylla *EGFR* test at the BC Cancer. The trial cohort included all stage IIIB/IV lung adenocarcinoma (AC) patients with a BC Cancer NGS molecular testing request between November 1, 2020, and May 1, 2021, with at least 500 ng of total DNA extracted from the formalin-fixed paraffin-embedded (FFPE) block so that both an Idylla *EGFR* test and NGS could be performed simultaneously. Having at least 500 ng of DNA also allows for confirmatory testing in cases with a discrepant result between Idylla and NGS. Patients that had insufficient tumor content (<20%) for NGS testing were excluded from the study, which was either due to limited tumor based on histological assessment or limited DNA post-extraction (less than 500 ng). The control cohort included all lung AC patients who received an NGS panel between August 1, 2020, and October 31, 2020. Patients who did not receive treatment or received treatment unrelated to molecular testing were not included in the analysis for TTT. 114 patients in both the control and trial cohorts had sufficient information for TTT analysis. 

This study was granted ethical approval by the UBC BC Cancer Research Ethics Board (REB #H20-03189, approved 16 December 2020) before study initiation.

### 2.2. Nucleic Acid Extraction

One slide was stained with H&E and evaluated by a Molecular Pathologist to check for tissue cellularity and tumor content and to identify the area for coring. Two to four cores of 1 mm in diameter were extracted from the tissue block for nucleic acid extraction. Briefly, 1 mL of hexadecane was added to the core in a 2 mL round bottom tube and incubated at 56 °C for 10 min. The cores were subjected to Tissuelyser for 2 min at 50 Hz using a 7 mm stainless steel ball, and then centrifuged to pellet cellular debris. The hexadecane was removed, and 1.6 mL of 100% ethanol was added. The sample was centrifuged again to pellet the cellular debris, and ethanol was removed. The sample was then vacuum-desiccated to dryness. The dry cellular debris was resuspended in 250 μL of lysis buffer (ref MC118A) plus proteinase K (Maxwell RSC DNA FFPE kit ref AS1450 Promega Biotechnology, Madison, WI, USA). The sample was digested overnight and then loaded onto the Maxwell RSC 48 (Promega Biotechnology, Madison, WI, USA) for DNA extraction. 

### 2.3. Idylla EGFR Mutation Testing

Single-gene mutation analysis for *EGFR* was performed on the Idylla platform using the EGFR cartridge, following the manufacturer’s protocol. The Idylla™ (Biocartis, Mechelen, Belgium) platform is a fully automated, real-time-PCR-based molecular testing system where all the reagents required for sample preparation and PCR detection are provided in a single cartridge that can then be loaded onto the system. The Idylla *EGFR* cartridge was loaded directly with 10 μL of DNA that had been diluted to 10 ng/μL (100 ng total load). The EGFR single-use cartridge, which is only compatible with the Idylla platform, performs qPCR to detect 51 mutations in exons 18 to 21 of *EGFR*. This assay is validated in the BC Cancer Genetics and Genomics Laboratory (CGL) for clinical reporting. 

### 2.4. Oncopanel NGS Testing

Subsequently, 100 ng of genomic DNA was subjected to Oncopanel NGS testing. Genomic DNA underwent FFPE repair, ligation-based library construction, PCR amplification, hybridization capture, and Illumina sequencing. Single-strand consensus sequences were generated from UMI-indexed reads using fgbio and aligned to the GRCh37 human genome reference using BWA. Variant calling was performed using samtools and VarScan2. Annotation and filtering of variants was performed with Agilent’s Alissa Interpret platform. The Oncopanel is designed to detect single-base and small insertion/deletion variants in the coding exonic sequence and at least 2 bp of flanking intronic sequence; or in known hotspots of clinical importance for 54 genes. The minimum depth of coverage required for reporting is 200×. Additional details, including a list of genes covered can be found at http://cancergeneticslab.ca (accessed on 13 July 2022). The sensitivity of the assay is deemed to be approximately 5% (site-specific analyses) to 10% (mutation screening). Variants present in less than this proportion of assayed alleles were not reported. Variants were interpreted in the context of available clinical information and tiered according to their known or predicted clinical significance (adapted from Li et al. [28]). 

### 2.5. Design Overview 

All data were collected through patient chart auditing. Data collected for patients in the control cohort included the time their sample reached the genetic testing lab, the result and date of the Oncopanel report, the time of first treatment, and the treatment regimen. In addition to the data collected for the control cohort, the result and date of the Idylla report was collected for the prospective cohort. Results of the Idylla test were compared with the results of the Oncopanel within the prospective cohort to validate the results of the Idylla *EGFF* test. The differences in lab TAT between Idylla and Oncopanel were also compared within the prospective cohort using a paired t-test to minimize confounding results that would have influenced lab TAT. 

TTT is measured from the time at which a biopsy is received in the lab to the time at which the patient receives their first treatment, for this instance of lung cancer. TTT was compared between the trial and control cohorts. Analysis of TTT was performed on groups of patients stratified based on whether they received treatment as *EGFR*-positive, PD-L1 ≥ 50%, neither *EGFR*-positive nor PD-L1 ≥ 50%, and overall. Patients were stratified based on being treated based on PD-L1 ≥ 50% because oncologists may begin treatment prior to Oncopanel results if there is a low pre-test probability of having an oncologic driver that will cause major side effects if switched from immunotherapy to TKI. The stratified cohorts were analyzed using a two-sample t-test or Mann–Whitney U test as appropriate.

### 2.6. Visualization

**Figure 1:**
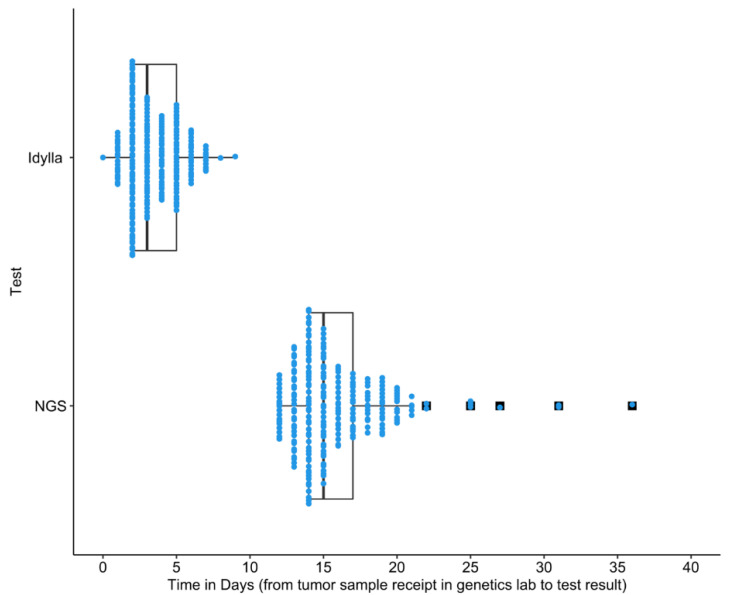
Comparison of laboratory TAT between the Idylla EGFR and Oncopanel NGS in patients who received concurrent Idylla and Oncopanel testing (N = 235). Each blue dot represents an individual at a certain time-point. Boxplots visualize median, two hinges (25% and 75% quantiles), and two whiskers (95% CI). Black squares indicate outliers. The difference in means was −12.4 days (*p* < 0.0001, 95% CI: [−12.8, −11.9] days). The mean lab TATs are 15.8 and 3.4 days for the Oncopanel and Idylla EGFR test, respectively.

**Figure 2:**
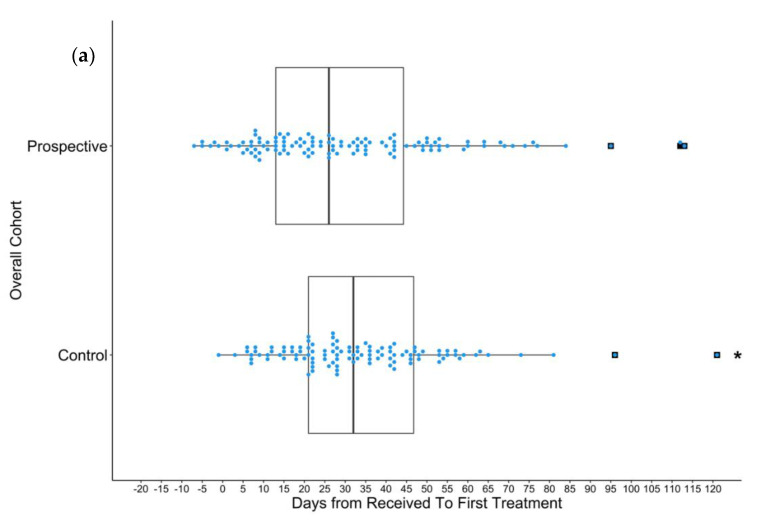

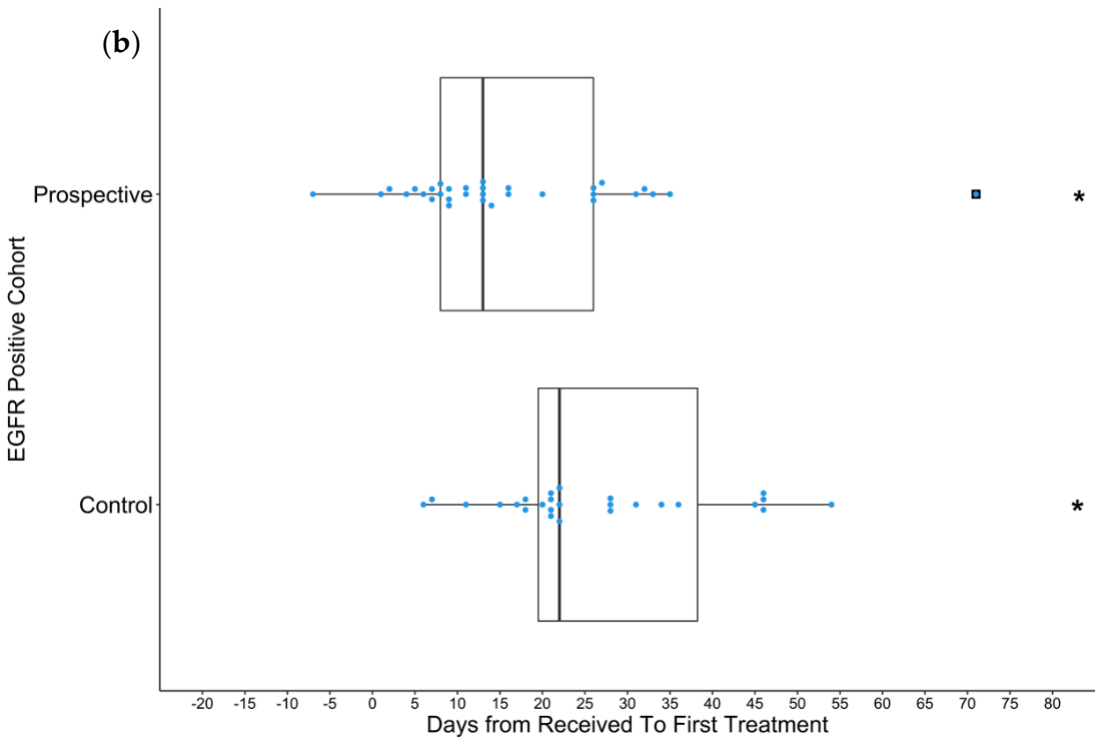
Boxplots visualize median, two hinges (25% and 75% quantiles), and two whiskers (95% CI). Black squares indicate outliers. Each blue dot represents an individual at a certain time-point. (**a**) The distribution of TTT of all patients in the control (N = 114) and trial (N = 114) cohorts who were included in TTT analysis. For the prospective trial cohort, the mean and median are 30.7 and 26 days, respectively. For the control cohort, the mean and median are 40.8 and 32 days, respectively. The difference in means is −10.1 days (*p* = 0.013, 95% CI: [−17.9, −2.2] days). (**b**) Distribution of TTT in patients who were treated based on EGFR positivity in the control (N = 28) and trial (N = 33) cohorts. For the prospective trial cohort, the mean and median are 18.5 and 13 days, respectively. For the control cohort, the mean and median are 35.3 and 22 days, respectively. Difference in means is −16.8 days (*p* = 0.03, 95% CI: [−32.1, −1.5] days). Asterisks indicate histograms with excluded data points.

**Figure 3:**
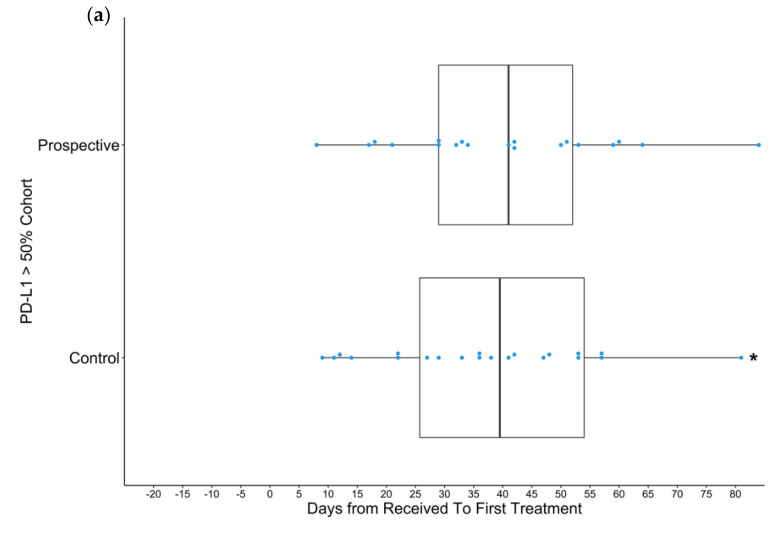

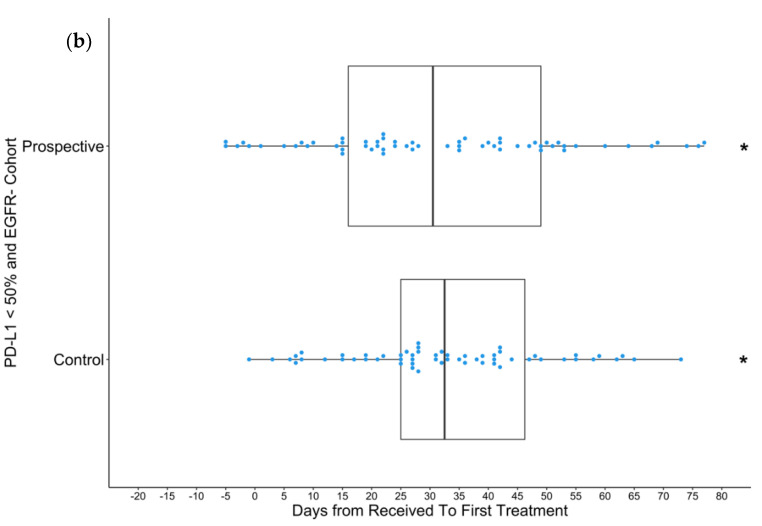
Boxplots visualize median, two hinges (25% and 75% quantiles), and two whiskers (95% CI). Black squares indicate outliers. Each blue dot represents an individual at a certain time-point. (**a**) The distribution of TTT of all patients treated based on PDL1 ≥ 50% in the control (N = 24) and trial (N = 19) cohorts. For the prospective trial cohort, the mean and median are 40.4 and 41 days, respectively. For the control cohort, the mean and median are 49.8 and 39.5 days, respectively. There is no statistically significant difference in means or medians. (**b**) The distribution of TTT of patients who received treatment based on PDL1 negativity and EGFR negativity in the control (N = 62) and trial (N = 62) cohorts. For the prospective trial cohort, the mean and median are 34.3 and 30.5 days, respectively. For the control cohort, the mean and median are 39.7 and 32.5 days, respectively. There is no statistically significant difference in means or medians. Asterisks indicate histograms with excluded data points.

Figure 1 includes all data points. The data ranges of the results presented in Figure 2 and Figure 3 were limited to improve clarity for comparison of distributions. As a result, outliers were excluded. From the overall control group, eight patients were excluded because their TTT exceeded 120 days (Figure 2a). Two patients in the control *EGFR+* control group and one in the prospective *EGFR+* group exceeded 80 days and were excluded (Figure 2b). Three patients in the control PD-L1 ≥ 50% group were excluded from because they exceeded a TTT of 80 days (Figure 3a). Four patients in the control non-PD-L1 ≥ 50%, non-*EGFR+* group and two patients in the prospective PD-L1 < 50% and *EGFR*-group exceeded 80 days and were excluded.

## 3. Results

High-level characteristics and outcomes are detailed in Supplementary Table S1.

In three cases (3/238), the NGS failed due to low coverage; thus, these patients were not included in the concordance comparison. A concordance frequency of 98.7% (232/235) was observed between the Idylla EGFR test and the NGS EGFR result (Table 1). Discrepant results were observed in two cases because of rare mutations that the Idylla EGFR test is not designed to detect. In one case, while both the Idylla EGFR test and Oncopanel correctly detected an actionable mutation, unlike the Idylla test, the Oncopanel did not report this mutation because the VAF for the variant was below the limit of detection (LOD). Raw data are included in Supplementary Table S2. The EGFR positivity rate observed in the control and trial cohorts were 17.7% (39/220) and 19.3% (46/238), respectively.

The lab TAT of the Idylla EGFR test was faster by an average of 12.4 days (*p* < 0.0001, 95% CI: [−12.8, −11.9] days) than the Oncopanel lab TAT (N = 235) (Figure 1). The mean lab TAT for the Idylla and Oncopanel tests were 3.4 and 15.8 days, respectively.

Overall, the average TTT in the trial cohort (N = 114) was 30.7 days, whereas the control (N = 114) cohort was 40.8 (Difference = 10.1 days, *p* < 0.05, 95% CI: [−17.9, −2.2] days), a 25% reduction in TTT (Figure 2a). In the trial cohort, most patients had a TTT of approximately 10 days (Figure 1). For patients who were treated based on EGFR positivity, the mean TTT was 18.5 days in the trial cohort (N = 33) and 35.3 days in the control cohort (N = 28) (Difference = 16.8 days, *p* < 0.05, 95% CI: [−32.1, −1.5] days), a 48% reduction in TTT (Figure 2b). Similar to the overall cohort, a large proportion of the EGFR-mutation-positive patients had a TTT of approximately 10 days. No significant difference in TTT (*p* = 0.89) was observed between patients treated based on PD-L1 ≥ 50% in the trial (N = 19) and control (N = 24) cohorts (Figure 3a). In the control PD-L1-high treatment cohort, 17% (4/24) of patients received treatment prior to obtaining EGFR mutation status and had the lowest TTT in the cohort (ranging from 9 to 14 days). However, no patients in the trial cohort received treatment prior to knowing EGFR mutation statuses. For patients who were treated based on being EGFR- and PD-L1-negative, there was also no significant difference in TTT between the trial (N = 62) and control (N = 62) cohorts (Figure 3b). The mean TTT for patients that were both EGFR-negative and not PD-L1-high was 34.3 days in the trial cohort and 39.7 days in the control cohort. Outliers were removed from Figure 2 and Figure 3, as described in the Methods section.

In the overall trial cohort, 31 patients received treatment after receiving the Idylla test result but before the Oncopanel result, with an average TTT savings of 7.6 days. In 52% (17/33) of EGFR-positive patients, treatment was initiated after receiving the Idylla test result but before the Oncopanel result. The TTT was 8.2 days on average for these patients. In patients who had a PD-L1 TPS of ≥ 50%, only 5% (1/19) had treatment initiated after the Idylla test result and before the Oncopanel results.

## 4. Discussion

These results shed light on the possibility of using an ultra-rapid *EGFR* test to improve TTT in advanced-stage NSCLC patients, and thus, patient outcomes. Our findings of a 98.7% concordance frequency between the Idylla *EGFR* test and the Oncopanel adheres with the previously established concordant rates of 94% to 100% [19,20,21,22,23,24,25,26]. It is of clinical significance that each discrepant result between the Idylla and the Oncopanel is not the result of random error in Idylla testing. This suggests that in cases where the Idylla *EGFR* test is positive, no further genetic testing would be required because all positive Idylla results were correct and *EGFR* mutations are, in practice, mutually exclusive with other oncogenic drivers [14,15,16,17,18]. Only in cases where Idylla *EGFR* mutation is negative would Oncopanel testing be required, for two specific reasons. First, the Idylla *EGFR* test covers a limited variety of *EGFR* mutations and does not detect rarer *EGFR* mutations that would otherwise be detected by more comprehensive *EGFR* gene coverage (performed by NGS testing). Second, it is important to perform genetic testing to check for other targetable oncogenic drivers if *EGFR* is negative. The *EGFR* mutation rate of the Canadian NSCLC population is about 15%; however, we observed an overall EGFR positivity rate that is closer to 20% [8,9]. This higher rate may be attributed to higher proportion of certain ethnicities in the B.C. population [6,7].

The lower laboratory TAT for ultra-rapid *EGFR* testing as compared with the Oncopanel allows for a faster completion of molecular testing in *EGFR*-positive patients. Our data demonstrate that the Idylla test was, on average, 12 days faster than the NGS Oncopanel results, which is similar to the finding of a study by Petiteau et al. where Idylla EGFR testing was, on average, 9 days faster than NGS [27]. Therefore, in about 30% of patients, genetic testing can be completed at a similar speed as testing by IHC, and does not act as a bottleneck for treatment [5]. Although most patients would be expected to be negative for *EGFR* mutations, the pre-test probability of harbouring an *EGFR*-mutation-positive tumor is higher in certain patient populations such as non-smokers. The true value of ultra-rapid *EGFR* testing in these patient subsets is the prompt identification of a targetable mutation and subsequent quick match to the appropriate treatment.

The use of one common source of tumor material for both tests by means of evaluating extracted DNA allowed for a side-by-side comparison of both assays. This would not be possible in instances where NGS testing is frequently conducted on extracted material while Idylla testing is performed directly on FFPE tissue. This also helps quantify and determine optimal Idylla load amounts based on tumor content and cellularity. Furthermore, this enables best triage of tumor material to the appropriate assays without the risk of under- or overloading.

Our findings of an improved TTT using ultra-rapid *EGFR* testing are an important addition to the limited body of literature on this topic. There is an impactful decrease in TTT in the *EGFR*-mutation-positive patients, a population that makes up a large portion of advanced-stage NSCLC patients [5]. It is likely that the large decrease in TTT observed in *EGFR*-mutation-positive patients is what drove the decrease in TTT in the overall cohort because, in both cases, a larger density of patients has a TTT of approximately 10 days in the trial group. The decrease in TTT has the potential to improve patient outcomes but further studies on this topic are warranted. Furthermore, the high proportion (17/33) of patients in the trial group who received EGFR-TKI treatment with genetic results only from the Idylla test, implies that many oncologists trust the result of the Idylla test and consider an *EGFR* single-gene test sufficient to initiate treatment. However, when patients were EGFR-negative, rarely did clinicians treat without Oncopanel results (14/205). It was expected that the patients who were treated based on being PD-L1-high would have a lower TTT if they had received Idylla *EGFR* testing; however, this was not observed. The oncologists treating patients in the trial cohort were likely waiting for the Oncopanel results for other oncogenic drivers prior to initiating treatment. This is reflected in that only one (1/19) of the PD-L1-high patients in the trial cohort were treated before Oncopanel results were received. Another reason for this observation may be that unlike EGFR-TKI, which is a per oral treatment that patients can begin immediately after a treatment decision is made, initiation of immunotherapy relies on hospital scheduling for IV administration and has more factors affecting TTT. Despite a homologous TTT between the trial and control groups in our study, using the Idylla *EGFR* test can rule out treating *EGFR*-mutation-positive patients with immunotherapy. This is critical because there can be severe consequences of administering immunotherapy in *EGFR*-mutation-positive patients due to the suboptimal efficacy of immunotherapy in this population and the toxicities associated with switching to *EGFR* TKI treatment [12,13,29,30,31]. Similarly, patients who were not PD-L1-high and *EGFR*-negative did not benefit from Idylla *EGFR* testing as these cases would follow the routine workflow. Since there are still other targetable alterations that could be detected by the Oncopanel, it is likely that clinicians found it best to wait for Oncopanel results prior to starting therapy.

There is value to a tiered-testing approach in a setting where a centralized genomics laboratory does mutational testing for lung cancer from many different sites or hospitals. As outlined, ultra-rapid *EGFR* assays help quickly identify patients that can start on targeted therapy without significantly altering testing workflow. For the patients that tested negative by single-gene testing, a more comprehensive testing strategy can be pursued from the same source tumor material. Alternatively, prompt single-gene tests can be performed at the originating sites/hospitals and those that tested negative can then be sent over to the central lab for additional/expanded testing. Both strategies could theoretically lead to faster and cheaper lung cancer molecular testing on average, with potential TTT savings. Based on our results, benefits would most likely be in EGFR-positive patients. Nevertheless, if comprehensive testing is delayed while waiting for *EGFR* single-gene testing results, *EGFR*-negative patients may have a longer TTT. The main pitfall of this approach is that in many instances for lung cancer specimens, the source material is scant permitting only one test. In this scenario, it may be prudent to do an assay with broader coverage so that material is not depleted by attempting the ultra-rapid *EGFR* assay.

## 5. Limitations and Future Directions

When stratifying patients to analyze TTT, the sample sizes of the *EGFR*-positive and PD-L1 ≥ 50% cohorts were small. While oncologists use patient demographics to determine the likelihood of a patient being *EGFR*-mutation-positive to expedite treatment, this study did not include patient demographics as part of the analysis. Our study utilized a historical control group, causing our comparison to be prone to confounding factors such as change in clinical or laboratory practices. Additionally, cohorts were studied during the COVID-19 pandemic; therefore, outcomes may have been impacted by policy changes at our institution.

Future studies may investigate how the improved TTT affects patient outcomes. Similar studies should be conducted on different oncogenic drivers as TKI treatment becomes available for more NSCLC-causing mutations. More research is needed to analyze the cost-effectiveness of using Idylla single-gene testing. Each cartridge of the Idylla EGFR test is expensive, but with certain workflows it may save costs.

## 6. Conclusions

Using the Idylla platform for ultra-rapid *EGFR* testing as part of the molecular testing repertoire in advanced-stage NSCLC patients with lung AC reduced their TTT. Genetic testing with the Idylla *EGFR* test may be sufficient if a positive result is obtained. Follow-up testing by a more expansive panel is needed if negative. Furthermore, this study demonstrates that, on average, TTT from molecular testing initiation can be reduced by 48% in *EGFR*-positive NSCLC patients. As cartridge-based molecular testing expands to hotspots in other genes and targets, a large population of NSCLC patients may see a meaningful reduction in TTT. This study shows how this paradigm could work for *EGFR* mutations, the most prevalent actionable biomarker.

## Figures and Tables

**Table 1:** Comparison of Idylla results to the standard Oncopanel NGS within the trial cohort. * Oncopanel did not report an *EGFR* exon 19 indel c.2236_2250delGAATTAAGAGAAGCA. The variant was present in NGS data at a VAF < 1% (below the LOD). ^ Idylla did not detect the *EGFR* exon 19 indel c.2239_2260delinsAGCCAAC (NGS VAF = 29%) or the *EGFR* exon 20 ins/dup c.2317delinsTACAACCCCT (NGS VAF = 25.1%) as these variants are not specifically targeted by the assay Three Oncopanel samples were reported as ‘Fail’ due to low coverage.

Idylla Results	Idylla Result Count	Oncopanel Agreement Count	Oncopanel Disagreement Results and Count	
G719X (Exon 18)	3	3	No Disagreements	
L858R	21	19	FAIL 2	
EXON 19 DEL	18	17	EGFR NEG * 1	
EXON 20 INS	2	2	No Disagreements	
EGFR NEG	194	191	EXON 20 INS/DUP ^	1
			EXON 19 INDEL ^	1
			FAIL	1

## Data Availability

Not applicable.

## Supplementary Materials

The following supporting information can be downloaded at: https://www.mdpi.com/article/10.3390/curroncol29100624/s1, Table S1: High level overview of trial characteristics and outcomes of the study, Table S2: Raw data of Idylla and Oncopanel EGFR test results and their respective Lab TAT.
