# Supplementary material for: Improving Time-to-Treatment for Advanced Non-Small Cell Lung Cancer Patients through Faster Single Gene EGFR Testing Using the Idylla™ EGFR Testing Platform
Source: Curr Oncol. 2022 Oct 18;29(10):7900–11. doi: 10.3390/curroncol29100624 (PMC9600153; doi:10.3390/curroncol29100624)
Supplement: Supplementary file 1 [file curroncol-29-00624-s001.zip › curroncol-1736485-supplementary.pdf]

Supplementary Files

**Table S1:** High level overview of trial characteristics and outcomes of the study. *Statistically significant ($p < 0.05$) comparison between control and prospective cohort.

| Parameter | Control Cohort | Prospective Cohort |
|---|---|---|
| Type of Molecular Testing Administered | NGS Oncopanel | NGS Oncopanel<br>Idylla EGFR |
| Overall Objective of Cohort | To act as a control for a baseline TTT with only NGS testing of EGFR | To investigate TTT with Idylla EGFR and Oncopanel testing. |
| Number of Patients in Cohort, N | 220 | 238 |
| EGFR positive patients, n | 39 | 46 |
| Concordance Frequency between Idylla and Oncopanel EGFR result, % (proportion) | NA | 98.7%<br>(232/235) |
| Laboratory TAT, mean (range) | Oncopanel: 14.5 (11, 27)<br>Idylla: NA | Oncopanel: 15.8 (12, 36)<br>Idylla: 3.4 (0, 9) |
| TTT in Overall Cohort, mean* | 40.8 days | 30.7 days |
| TTT in EGFR positive patients, mean* | 35.3 days | 18.5 days |

**Table S2:** Raw data of Idylla and Oncopanel EGFR test results and their respective Lab TAT.

| | | Idylla | | | Oncopanel | | |
|---|---|---|---|---|---|---|---|
| Patient Number | Specimen Received in Lab | Idylla Report Date | Idylla Result | Idylla Lab TAT | Oncopanel Report Date | Oncopanel EGFR RESULT | Oncopanel Lab TAT |
| 1 | 2-Nov-20 | 6-Nov-20 | NEGATIVE | 4 | 17-Nov-20 | EGFR NEG WITH OTHER POS | 15 |
| 2 | 6-Nov-20 | 9-Nov-20 | NEGATIVE | 3 | 20-Nov-20 | EGFR NEG WITH OTHER POS | 14 |
| 3 | 6-Nov-20 | 9-Nov-20 | NEGATIVE | 3 | 19-Nov-20 | NO REPORTABLE VARIANTS | 13 |
| 4 | 9-Nov-20 | 10-Nov-20 | NEGATIVE | 1 | 24-Nov-20 | EGFR NEG WITH OTHER POS | 15 |
| 5 | 9-Nov-20 | 13-Nov-20 | NEGATIVE | 4 | 24-Nov-20 | EGFR NEG WITH OTHER POS | 15 |
| 6 | 9-Nov-20 | 10-Nov-20 | L858R | 1 | 24-Nov-20 | L858R (VAF: 19.1%) | 15 |
| 7 | 9-Nov-20 | 10-Nov-20 | NEGATIVE | 1 | 24-Nov-20 | EGFR NEG WITH OTHER POS | 15 |
| 8 | 10-Nov-20 | 16-Nov-20 | NEGATIVE | 6 | 26-Nov-20 | EGFR NEG WITH OTHER POS | 16 |
| 9 | 10-Nov-20 | 16-Nov-20 | NEGATIVE | 6 | 26-Nov-20 | EGFR NEG | 16 |
| 10 | 10-Nov-20 | 16-Nov-20 | NEGATIVE | 6 | 26-Nov-20 | EGFR NEG WITH OTHER POS | 16 |
| 11 | 12-Nov-20 | 16-Nov-20 | NEGATIVE | 4 | 26-Nov-20 | EGFR NEG WITH OTHER POS | 14 |
| 12 | 12-Nov-20 | 16-Nov-20 | NEGATIVE | 4 | 26-Nov-20 | EGFR NEG WITH OTHER POS | 14 |
| 13 | 12-Nov-20 | 16-Nov-20 | NEGATIVE | 4 | 1-Dec-20 | EGFR NEG WITH OTHER POS | 19 |
| 14 | 13-Nov-20 | 16-Nov-20 | NEGATIVE | 3 | 26-Nov-20 | EGFR NEG WITH OTHER POS | 13 |
| 15 | 16-Nov-20 | 18-Nov-20 | NEGATIVE | 2 | 3-Dec-20 | EGFR NEG WITH OTHER POS | 17 |
| 16 | 16-Nov-20 | 18-Nov-20 | X19 DELETION | 2 | 3-Dec-20 | X19 DELETION (VAF: 13.3%) | 17 |
| 17 | 16-Nov-20 | 18-Nov-20 | NEGATIVE | 2 | 3-Dec-20 | EGFR NEG WITH OTHER POS | 17 |
| 18 | 16-Nov-20 | 19-Nov-20 | NEGATIVE | 3 | 3-Dec-20 | EGFR NEG WITH OTHER POS | 17 |
| 19 | 16-Nov-20 | 19-Nov-20 | NEGATIVE | 3 | 4-Dec-20 | EGFR NEG WITH OTHER POS | 18 |
| 20 | 16-Nov-20 | 19-Nov-20 | NEGATIVE | 3 | 3-Dec-20 | EGFR NEG WITH OTHER POS | 17 |

| | | | | | | | | |
|---|---|---|---|---|---|---|---|---|
| 21 | 19-Nov-20 | 20-Nov-20 | NEGATIVE | 1 | 1-Dec-20 | EGFR NEG WITH OTHER POS | 12 |
| 22 | 20-Nov-20 | 23-Nov-20 | NEGATIVE | 3 | 4-Dec-20 | EGFR NEG WITH OTHER POS | 14 |
| 23 | 20-Nov-20 | 23-Nov-20 | X19 DELETION | 3 | 4-Dec-20 | X19 DELETION (VAF: 16.4%) | 14 |
| 24 | 20-Nov-20 | 23-Nov-20 | NEGATIVE | 3 | 4-Dec-20 | EGFR NEG WITH OTHER POS | 14 |
| 25 | 20-Nov-20 | 23-Nov-20 | NEGATIVE | 3 | 4-Dec-20 | EGFR NEG | 14 |
| 26 | 20-Nov-20 | 24-Nov-20 | NEGATIVE | 4 | 4-Dec-20 | EGFR NEG WITH OTHER POS | 14 |
| 27 | 20-Nov-20 | 24-Nov-20 | NEGATIVE | 4 | 4-Dec-20 | EGFR NEG WITH OTHER POS | 14 |
| 28 | 23-Nov-20 | 25-Nov-20 | NEGATIVE | 2 | 9-Dec-20 | EGFR NEG WITH OTHER POS | 16 |
| 29 | 23-Nov-20 | 25-Nov-20 | NEGATIVE | 2 | 9-Dec-20 | EGFR NEG WITH OTHER POS | 16 |
| 30 | 24-Nov-20 | 25-Nov-20 | NEGATIVE | 1 | 9-Dec-20 | EGFR NEG WITH OTHER POS | 15 |
| 31 | 24-Nov-20 | 27-Nov-20 | NEGATIVE | 3 | 9-Dec-20 | EGFR NEG WITH OTHER POS | 15 |
| 32 | 27-Nov-20 | 1-Dec-20 | NEGATIVE | 4 | 9-Dec-20 | EGFR NEG WITH OTHER POS | 12 |
| 33 | 1-Dec-20 | 4-Dec-20 | NEGATIVE | 3 | 16-Dec-20 | EGFR NEG WITH OTHER POS | 15 |
| 34 | 1-Dec-20 | 3-Dec-20 | NEGATIVE | 2 | 16-Dec-20 | EGFR NEG WITH OTHER POS | 15 |
| 35 | 1-Dec-20 | 3-Dec-20 | NEGATIVE | 2 | 16-Dec-20 | EGFR NEG WITH OTHER POS | 15 |
| 36 | 1-Dec-20 | 4-Dec-20 | L858R | 3 | 16-Dec-20 | L858R (VAF: 87.0%) | 15 |
| 37 | 2-Dec-20 | 4-Dec-20 | NEGATIVE | 2 | 16-Dec-20 | EGFR NEG WITH OTHER POS | 14 |
| 38 | 2-Dec-20 | 7-Dec-20 | NEGATIVE | 5 | 16-Dec-20 | EGFR NEG WITH OTHER POS | 14 |
| 39 | 3-Dec-20 | 7-Dec-20 | NEGATIVE | 4 | 16-Dec-20 | EGFR NEG WITH OTHER POS | 13 |
| 40 | 3-Dec-20 | 7-Dec-20 | NEGATIVE | 4 | 16-Dec-20 | EGFR NEG WITH OTHER POS | 13 |
| 41 | 4-Dec-20 | 7-Dec-20 | NEGATIVE | 3 | 16-Dec-20 | EGFR NEG WITH OTHER POS | 12 |
| 42 | 4-Dec-20 | 8-Dec-20 | G719A | 4 | 22-Dec-20 | G719A (VAF: 29.6%) | 18 |
| 43 | 4-Dec-20 | 8-Dec-20 | X19 DELETION | 4 | 22-Dec-20 | X19 DELETION (VAF: 17.2%) | 18 |
| 44 | 4-Dec-20 | 8-Dec-20 | NEGATIVE | 4 | 22-Dec-20 | EGFR NEG WITH OTHER POS | 18 |
| 45 | 7-Dec-20 | 8-Dec-20 | NEGATIVE | 1 | 22-Dec-20 | EGFR NEG WITH OTHER POS | 15 |
| 46 | 7-Dec-20 | 9-Dec-20 | NEGATIVE | 2 | 22-Dec-20 | EGFR NEG WITH OTHER POS | 15 |
| 47 | 7-Dec-20 | 9-Dec-20 | NEGATIVE | 2 | 22-Dec-20 | EGFR NEG WITH OTHER POS | 15 |
| 48 | 7-Dec-20 | 9-Dec-20 | NEGATIVE | 2 | 22-Dec-20 | EGFR NEG WITH OTHER POS | 15 |
| 49 | 7-Dec-20 | 10-Dec-20 | NEGATIVE | 3 | 22-Dec-20 | EGFR NEG WITH OTHER POS | 15 |
| 50 | 7-Dec-20 | 10-Dec-20 | NEGATIVE | 3 | 22-Dec-20 | EGFR NEG WITH OTHER POS | 15 |
| 51 | 7-Dec-20 | 10-Dec-20 | NEGATIVE | 3 | 22-Dec-20 | EGFR NEG WITH OTHER POS | 15 |
| 52 | 8-Dec-20 | 10-Dec-20 | NEGATIVE | 2 | 22-Dec-20 | EGFR NEG WITH OTHER POS | 14 |
| 53 | 8-Dec-20 | 10-Dec-20 | NEGATIVE | 2 | 22-Dec-20 | EGFR NEG WITH OTHER POS | 14 |
| 54 | 8-Dec-20 | 14-Dec-20 | NEGATIVE | 6 | 22-Dec-20 | EGFR NEG WITH OTHER POS | 14 |
| 55 | 9-Dec-20 | 14-Dec-20 | NEGATIVE | 5 | 22-Dec-20 | EGFR NEG WITH OTHER POS | 13 |
| 56 | 9-Dec-20 | 14-Dec-20 | NEGATIVE | 5 | 23-Dec-20 | EGFR NEG WITH OTHER POS | 14 |
| 57 | 9-Dec-20 | 14-Dec-20 | NEGATIVE | 5 | 21-Dec-20 | EGFR NEG WITH OTHER POS | 12 |
| 58 | 9-Dec-20 | 14-Dec-20 | NEGATIVE | 5 | 21-Dec-20 | EGFR NEG WITH OTHER POS | 12 |

| | | | | | | | |
|---|---|---|---|---|---|---|---|
| 59 | 10-Dec-20 | 14-Dec-20 | #N/A | 4 | 23-Dec-20 | N/A | 13 |
| 60 | 10-Dec-20 | 14-Dec-20 | #N/A | 4 | 24-Dec-20 | N/A | 14 |
| 61 | 11-Dec-20 | 14-Dec-20 | NEGATIVE | 3 | 24-Dec-20 | EGFR NEG WITH OTHER POS | 13 |
| 62 | 11-Dec-20 | 14-Dec-20 | NEGATIVE | 3 | 23-Dec-20 | EGFR POS EXON 20 (VAF25%) | 12 |
| 63 | 11-Dec-20 | 14-Dec-20 | NEGATIVE | 3 | 23-Dec-20 | EGFR NEG WITH OTHER POS | 12 |
| 64 | 11-Dec-20 | 14-Dec-20 | NEGATIVE | 3 | 23-Dec-20 | EGFR NEG WITH OTHER POS | 12 |
| 65 | 14-Dec-20 | 15-Dec-20 | NEGATIVE | 1 | 31-Dec-20 | EGFR NEG WITH OTHER POS | 17 |
| 66 | 14-Dec-20 | 16-Dec-20 | NEGATIVE | 2 | 31-Dec-20 | EGFR NEG WITH OTHER POS | 17 |
| 67 | 15-Dec-20 | 16-Dec-20 | NEGATIVE | 1 | 31-Dec-20 | EGFR NEG WITH OTHER POS | 16 |
| 68 | 15-Dec-20 | 17-Dec-20 | NEGATIVE | 2 | 31-Dec-20 | EGFR NEG WITH OTHER POS | 16 |
| 69 | 16-Dec-20 | 18-Dec-20 | NEGATIVE | 2 | 30-Dec-20 | EGFR NEG WITH OTHER POS | 14 |
| 70 | 18-Dec-20 | 21-Dec-20 | NEGATIVE | 3 | 31-Dec-20 | EGFR NEG WITH OTHER POS | 13 |
| 71 | 21-Dec-20 | 23-Dec-20 | NEGATIVE | 2 | 11-Jan-21 | EGFR NEG WITH OTHER POS | 21 |
| 72 | 21-Dec-20 | 22-Dec-20 | NEGATIVE | 1 | 11-Jan-21 | EGFR NEG | 21 |
| 73 | 21-Dec-20 | 22-Dec-20 | L858R | 1 | 11-Jan-21 | L858R (VAF: 93.2%) | 21 |
| 74 | 21-Dec-20 | 24-Dec-20 | NEGATIVE | 3 | 11-Jan-21 | EGFR NEG WITH OTHER POS | 21 |
| 75 | 22-Dec-20 | 24-Dec-20 | NEGATIVE | 2 | 11-Jan-21 | EGFR NEG WITH OTHER POS | 20 |
| 76 | 22-Dec-20 | 24-Dec-20 | NEGATIVE | 2 | 11-Jan-21 | EGFR NEG WITH OTHER POS | 20 |
| 77 | 23-Dec-20 | 28-Dec-20 | NEGATIVE | 5 | 11-Jan-21 | EGFR NEG WITH OTHER POS | 19 |
| 78 | 23-Dec-20 | 28-Dec-20 | NEGATIVE | 5 | 11-Jan-21 | EGFR NEG WITH OTHER POS | 19 |
| 79 | 23-Dec-20 | 28-Dec-20 | NEGATIVE | 5 | 11-Jan-21 | EGFR NEG WITH OTHER POS | 19 |
| 80 | 23-Dec-20 | 28-Dec-20 | NEGATIVE | 5 | 12-Jan-21 | EGFR NEG WITH OTHER POS | 20 |
| 81 | 23-Dec-20 | 28-Dec-20 | NEGATIVE | 5 | 11-Jan-21 | EGFR NEG WITH OTHER POS | 19 |
| 82 | 23-Dec-20 | 28-Dec-20 | NEGATIVE | 5 | 11-Jan-21 | EGFR NEG WITH OTHER POS | 19 |
| 83 | 23-Dec-20 | 28-Dec-20 | NEGATIVE | 5 | 12-Jan-21 | EGFR NEG WITH OTHER POS | 20 |
| 84 | 24-Dec-20 | 31-Dec-20 | NEGATIVE | 7 | 18-Jan-21 | EGFR NEG WITH OTHER POS | 25 |
| 85 | 24-Dec-20 | 31-Dec-20 | NEGATIVE | 7 | 18-Jan-21 | EGFR NEG WITH OTHER POS | 25 |
| 86 | 24-Dec-20 | 31-Dec-20 | NEGATIVE | 7 | 29-Jan-21 | EGFR NEG WITH OTHER POS | 36 |
| 87 | 29-Dec-20 | 31-Dec-20 | NEGATIVE | 2 | 20-Jan-21 | EGFR NEG | 22 |
| 88 | 29-Dec-20 | 31-Dec-20 | NEGATIVE | 2 | 20-Jan-21 | EGFR NEG WITH OTHER POS | 22 |
| 89 | 29-Dec-20 | 31-Dec-20 | NEGATIVE | 2 | 18-Jan-21 | EGFR NEG WITH OTHER POS | 20 |
| 90 | 29-Dec-20 | 31-Dec-20 | NEGATIVE | 2 | 18-Jan-21 | EGFR NEG | 20 |
| 91 | 29-Dec-20 | 4-Jan-21 | NEGATIVE | 6 | 18-Jan-21 | EGFR NEG WITH OTHER POS | 20 |
| 92 | 29-Dec-20 | 4-Jan-21 | NEGATIVE | 6 | 18-Jan-21 | EGFR NEG WITH OTHER POS | 20 |
| 93 | 30-Dec-20 | 4-Jan-21 | NEGATIVE | 5 | 18-Jan-21 | EGFR NEG WITH OTHER POS | 19 |
| 94 | 30-Dec-20 | 4-Jan-21 | NEGATIVE | 5 | 18-Jan-21 | EGFR NEG WITH OTHER POS | 19 |
| 95 | 30-Dec-20 | 4-Jan-21 | X19 DELETION | 5 | 18-Jan-21 | X19 DELETION (VAF: 33.0%) | 19 |
| 96 | 31-Dec-20 | 5-Jan-21 | NEGATIVE | 5 | 18-Jan-21 | EGFR NEG WITH OTHER POS | 18 |

| | | | | | | | |
|---|---|---|---|---|---|---|---|
| 97 | 31-Dec-20 | 5-Jan-21 | NEGATIVE | 5 | 18-Jan-21 | EGFR NEG WITH OTHER POS | 18 |
| 98 | 31-Dec-20 | 5-Jan-21 | NEGATIVE | 5 | 19-Jan-21 | EGFR NEG WITH OTHER POS | 19 |
| 99 | 4-Jan-21 | 7-Jan-21 | X19 DELETION | 3 | 18-Jan-21 | X19 DELETION (VAF: 26.9%) | 14 |
| 100 | 4-Jan-21 | 6-Jan-21 | NEGATIVE | 2 | 18-Jan-21 | EGFR NEG WITH OTHER POS | 14 |
| 101 | 4-Jan-21 | 6-Jan-21 | NEGATIVE | 2 | 18-Jan-21 | EGFR NEG WITH OTHER POS | 14 |
| 102 | 4-Jan-21 | 7-Jan-21 | L858R | 3 | 18-Jan-21 | L858R (VAF: 17.9%) | 14 |
| 103 | 4-Jan-21 | 7-Jan-21 | X19 DELETION | 3 | 18-Jan-21 | X19 DELETION (VAF: 29.3%) | 14 |
| 104 | 4-Jan-21 | 7-Jan-21 | NEGATIVE | 3 | 18-Jan-21 | EGFR NEG WITH OTHER POS | 14 |
| 105 | 6-Jan-21 | 8-Jan-21 | L858R | 2 | 21-Jan-21 | L858R (VAF: 8.0%) | 15 |
| 106 | 6-Jan-21 | 8-Jan-21 | X19 DELETION | 2 | 26-Jan-21 | ***NEGATIVE*** | 20 |
| 107 | 6-Jan-21 | 8-Jan-21 | X20 INS | 2 | 21-Jan-21 | X20 INS (VAF: 28.6%) | 15 |
| 108 | 6-Jan-21 | 8-Jan-21 | L858R | 2 | 25-Jan-21 | L858R (VAF: 54.9%) | 19 |
| 109 | 8-Jan-21 | 11-Jan-21 | NEGATIVE | 3 | 25-Jan-21 | NO REPORTABLE VARIANTS | 17 |
| 110 | 8-Jan-21 | 11-Jan-21 | NEGATIVE | 3 | 25-Jan-21 | EGFR NEG WITH OTHER POS | 17 |
| 111 | 11-Jan-21 | 13-Jan-21 | NEGATIVE | 2 | 28-Jan-21 | EGFR NEG WITH OTHER POS | 17 |
| 112 | 11-Jan-21 | 13-Jan-21 | NEGATIVE | 2 | 28-Jan-21 | No Reportable Variants/low coverage | 17 |
| 113 | 12-Jan-21 | 15-Jan-21 | NEGATIVE | 3 | 28-Jan-21 | EGFR NEG WITH OTHER POS | 16 |
| 114 | 12-Jan-21 | 15-Jan-21 | NEGATIVE | 3 | 28-Jan-21 | EGFR NEG WITH OTHER POS | 16 |
| 115 | 12-Jan-21 | 15-Jan-21 | NEGATIVE | 3 | 25-Jan-21 | EGFR NEG WITH OTHER POS | 13 |
| 116 | 12-Jan-21 | 15-Jan-21 | NEGATIVE | 3 | 27-Jan-21 | EGFR NEG WITH OTHER POS | 15 |
| 117 | 13-Jan-21 | 15-Jan-21 | NEGATIVE | 2 | 28-Jan-21 | EGFR NEG WITH OTHER POS | 15 |
| 118 | 19-Jan-21 | 20-Jan-21 | NEGATIVE | 1 | 2-Feb-21 | EGFR NEG WITH OTHER POS | 14 |
| 119 | 20-Jan-21 | 22-Jan-21 | NEGATIVE | 2 | 4-Feb-21 | EGFR NEG WITH OTHER POS | 15 |
| 120 | 20-Jan-21 | 22-Jan-21 | NEGATIVE | 2 | 4-Feb-21 | EGFR NEG WITH OTHER POS | 15 |
| 121 | 21-Jan-21 | 25-Jan-21 | NEGATIVE | 4 | 3-Feb-21 | EGFR NEG WITH OTHER POS | 13 |
| 122 | 21-Jan-21 | 21-Jan-21 | L858R | 0 | 3-Feb-21 | L858R (VAF: 25.2%) | 13 |
| 123 | 22-Jan-21 | 27-Jan-21 | L858R | 5 | 3-Feb-21 | L858R (VAF: 8.0%) | 12 |
| 124 | 22-Jan-21 | 25-Jan-21 | NEGATIVE | 3 | 3-Feb-21 | EGFR NEG WITH OTHER POS | 12 |
| 125 | 25-Jan-21 | 27-Jan-21 | NEGATIVE | 2 | 8-Feb-21 | EGFR NEG WITH OTHER POS | 14 |
| 126 | 25-Jan-21 | 28-Jan-21 | NEGATIVE | 3 | 9-Feb-21 | EGFR NEG WITH OTHER POS | 15 |
| 127 | 25-Jan-21 | 28-Jan-21 | G719 | 3 | 8-Feb-21 | G719 (VAF: 52.6%) + EXON 21 L861R (VAF: 51.1%) | 14 |
| 128 | 26-Jan-21 | 28-Jan-21 | NEGATIVE | 2 | 8-Feb-21 | EGFR NEG WITH OTHER POS | 13 |
| 129 | 26-Jan-21 | 1-Feb-21 | NEGATIVE | 6 | 16-Feb-21 | NO REPORTABLE VARIANTS | 21 |
| 130 | 26-Jan-21 | 28-Jan-21 | L858R | 2 | 8-Feb-21 | L858R (VAF: 50.9%) | 13 |
| 131 | 27-Jan-21 | 29-Jan-21 | NEGATIVE | 2 | 12-Feb-21 | EGFR NEG WITH OTHER POS | 16 |
| 132 | 27-Jan-21 | 29-Jan-21 | NEGATIVE | 2 | 15-Feb-21 | EGFR NEG WITH OTHER POS | 19 |
| 133 | 28-Jan-21 | 1-Feb-21 | NEGATIVE | 4 | 15-Feb-21 | EGFR NEG WITH OTHER POS | 18 |

| | | | | | | | |
|---|---|---|---|---|---|---|---|
| 134 | 28-Jan-21 | 1-Feb-21 | NEGATIVE | 4 | 15-Feb-21 | NO REPORTABLE VARIANTS | 18 |
| 135 | 29-Jan-21 | 3-Feb-21 | X19 DELETION | 5 | 15-Feb-21 | X19 DELETION (VAF: 10.4%) | 17 |
| 136 | 29-Jan-21 | 3-Feb-21 | X19 DELETION | 5 | 15-Feb-21 | X19 DELETION (VAF: 23.2%) | 17 |
| 137 | 29-Jan-21 | 3-Feb-21 | NEGATIVE | 5 | 15-Feb-21 | EGFR NEG WITH OTHER POS | 17 |
| 138 | 29-Jan-21 | 3-Feb-21 | NEGATIVE | 5 | 15-Feb-21 | EGFR NEG WITH OTHER POS | 17 |
| 139 | 1-Feb-21 | 3-Feb-21 | NEGATIVE | 2 | 19-Feb-21 | EGFR 2492G>A Arg831His (VAF 44.6%) | 18 |
| 140 | 1-Feb-21 | 3-Feb-21 | NEGATIVE | 2 | 17-Feb-21 | EGFR NEG WITH OTHER POS | 16 |
| 141 | 2-Feb-21 | 5-Feb-21 | NEGATIVE | 3 | 17-Feb-21 | EGFR NEG WITH OTHER POS | 15 |
| 142 | 2-Feb-21 | 3-Feb-21 | G719 | 1 | 17-Feb-21 | G719 (VAF 3.8%) | 15 |
| 143 | 2-Feb-21 | 4-Feb-21 | NEGATIVE | 2 | 19-Feb-21 | EGFR NEG WITH OTHER POS | 17 |
| 144 | 2-Feb-21 | 4-Feb-21 | NEGATIVE | 2 | 17-Feb-21 | EGFR NEG WITH OTHER POS | 15 |
| 145 | 2-Feb-21 | 4-Feb-21 | NEGATIVE | 2 | 17-Feb-21 | EGFR Leu747_LysdelinsSerGlnGln (VAF 29.0%) | 15 |
| 146 | 4-Feb-21 | 9-Feb-21 | NEGATIVE | 5 | 17-Feb-21 | EGFR NEG WITH OTHER POS | 13 |
| 147 | 4-Feb-21 | 9-Feb-21 | NEGATIVE | 5 | 17-Feb-21 | NO REPORTABLE VARIANTS | 13 |
| 148 | 5-Feb-21 | 9-Feb-21 | NEGATIVE | 4 | 17-Feb-21 | EGFR NEG WITH OTHER POS | 12 |
| 149 | 5-Feb-21 | 10-Feb-21 | NEGATIVE | 5 | 19-Feb-21 | EGFR NEG WITH OTHER POS | 14 |
| 150 | 5-Feb-21 | 10-Feb-21 | NEGATIVE | 5 | 19-Feb-21 | EGFR NEG WITH OTHER POS | 14 |
| 151 | 10-Feb-21 | 12-Feb-21 | NEGATIVE | 2 | 24-Feb-21 | EGFR NEG WITH OTHER POS | 14 |
| 152 | 10-Feb-21 | 12-Feb-21 | L858R | 2 | 24-Feb-21 | L858R (VAF: 16.0%) | 14 |
| 153 | 10-Feb-21 | 12-Feb-21 | X19 DELETION | 2 | 23-Feb-21 | X19 DELETION (VAF: 60.8%) | 13 |
| 154 | 11-Feb-21 | 12-Feb-21 | L858R | 1 | 23-Feb-21 | L858R (VAF: 29.5%) | 12 |
| 155 | 11-Feb-21 | 12-Feb-21 | NEGATIVE | 1 | 23-Feb-21 | EGFR NEG WITH OTHER POS | 12 |
| 156 | 11-Feb-21 | 12-Feb-21 | X19 DELETION | 1 | 23-Feb-21 | X19 DELETION (VAF: 14.9%) | 12 |
| 157 | 11-Feb-21 | 19-Feb-21 | NEGATIVE | 8 | 2-Mar-21 | EGFR NEG WITH OTHER POS | 19 |
| 158 | 11-Feb-21 | 16-Feb-21 | NEGATIVE | 5 | 23-Feb-21 | NO REPORTABLE VARIANTS | 12 |
| 159 | 12-Feb-21 | 16-Feb-21 | NEGATIVE | 4 | 24-Feb-21 | EGFR NEG WITH OTHER POS | 12 |
| 160 | 12-Feb-21 | 16-Feb-21 | NEGATIVE | 4 | 24-Feb-21 | EGFR NEG WITH OTHER POS | 12 |
| 161 | 16-Feb-21 | 17-Feb-21 | NEGATIVE | 1 | 2-Mar-21 | EGFR NEG WITH OTHER POS | 14 |
| 162 | 18-Feb-21 | 23-Feb-21 | NEGATIVE | 5 | 10-Mar-21 | EGFR NEG WITH OTHER POS | 20 |
| 163 | 18-Feb-21 | 23-Feb-21 | NEGATIVE | 5 | 3-Mar-21 | EGFR NEG WITH OTHER POS | 13 |
| 164 | 18-Feb-21 | 23-Feb-21 | NEGATIVE | 5 | 3-Mar-21 | EGFR NEG WITH OTHER POS | 13 |
| 165 | 19-Feb-21 | 23-Feb-21 | NEGATIVE | 4 | 4-Mar-21 | EGFR NEG WITH OTHER POS | 13 |
| 166 | 19-Feb-21 | 24-Feb-21 | L858R | 5 | 4-Mar-21 | L858R (VAF: 8.2%) | 13 |
| 167 | 19-Feb-21 | 24-Feb-21 | NEGATIVE | 5 | 4-Mar-21 | EGFR NEG WITH OTHER POS | 13 |
| 168 | 19-Feb-21 | 24-Feb-21 | NEGATIVE | 5 | 4-Mar-21 | EGFR NEG WITH OTHER POS | 13 |

| | | | | | | | |
|---|---|---|---|---|---|---|---|
| 169 | 22-Feb-21 | 24-Feb-21 | NEGATIVE | 2 | 10-Mar-21 | EGFR NEG WITH OTHER POS | 16 |
| 170 | 22-Feb-21 | 24-Feb-21 | NEGATIVE | 2 | 10-Mar-21 | EGFR NEG WITH OTHER POS | 16 |
| 171 | 22-Feb-21 | 24-Feb-21 | NEGATIVE | 2 | 10-Mar-21 | EGFR NEG WITH OTHER POS | 16 |
| 172 | 23-Feb-21 | 25-Feb-21 | X20 INS | 2 | 10-Mar-21 | X20 INS (VAF: 35.3%) | 15 |
| 173 | 23-Feb-21 | 1-Mar-21 | NEGATIVE | 6 | 10-Mar-21 | NO REPORTABLE VARIANTS | 15 |
| 174 | 23-Feb-21 | 1-Mar-21 | L858R | 6 | 11-Mar-21 | L858R (VAF: 30.2%) | 16 |
| 175 | 23-Feb-21 | 1-Mar-21 | NEGATIVE | 6 | 11-Mar-21 | EGFR NEG WITH OTHER POS | 16 |
| 176 | 23-Feb-21 | 1-Mar-21 | NEGATIVE | 6 | 11-Mar-21 | EGFR NEG WITH OTHER POS | 16 |
| 177 | 23-Feb-21 | 1-Mar-21 | NEGATIVE | 6 | 10-Mar-21 | EGFR NEG WITH OTHER POS | 15 |
| 178 | 23-Feb-21 | 1-Mar-21 | NEGATIVE | 6 | 11-Mar-21 | EGFR NEG WITH OTHER POS | 16 |
| 179 | 23-Feb-21 | 1-Mar-21 | X19 DELETION | 6 | 12-Mar-21 | X19 DELETION (VAF: 44.1%) | 17 |
| 180 | 23-Feb-21 | 1-Mar-21 | NEGATIVE | 6 | 11-Mar-21 | EGFR NEG WITH OTHER POS | 16 |
| 181 | 25-Feb-21 | 1-Mar-21 | NEGATIVE | 4 | 12-Mar-21 | EGFR NEG WITH OTHER POS | 15 |
| 182 | 25-Feb-21 | 1-Mar-21 | NEGATIVE | 4 | 11-Mar-21 | EGFR NEG WITH OTHER POS | 14 |
| 183 | 26-Feb-21 | 1-Mar-21 | NEGATIVE | 3 | 11-Mar-21 | EGFR NEG WITH OTHER POS | 13 |
| 184 | 1-Mar-21 | 4-Mar-21 | NEGATIVE | 3 | 17-Mar-21 | NO REPORTABLE VARIANTS | 16 |
| 185 | 2-Mar-21 | 4-Mar-21 | X19 DELETION | 2 | 17-Mar-21 | X19 DELETION (VAF: 50.5%) | 15 |
| 186 | 2-Mar-21 | 4-Mar-21 | NEGATIVE | 2 | 17-Mar-21 | EGFR NEG WITH OTHER POS | 15 |
| 187 | 3-Mar-21 | 4-Mar-21 | NEGATIVE | 1 | 17-Mar-21 | EGFR NEG WITH OTHER POS | 14 |
| 188 | 3-Mar-21 | 4-Mar-21 | NEGATIVE | 1 | 17-Mar-21 | EGFR NEG WITH OTHER POS | 14 |
| 189 | 3-Mar-21 | 4-Mar-21 | X19 DELETION | 1 | 17-Mar-21 | X19 DELETION (VAF: 6.0%) | 14 |
| 190 | 3-Mar-21 | 4-Mar-21 | NEGATIVE | 1 | 16-Mar-21 | EGFR NEG WITH OTHER POS | 13 |
| 191 | 4-Mar-21 | 10-Mar-21 | NEGATIVE | 6 | 22-Mar-21 | EGFR NEG WITH OTHER POS | 18 |
| 192 | 4-Mar-21 | 9-Mar-21 | L858R | 5 | 22-Mar-21 | L858R (VAF: 36.5%) | 18 |
| 193 | 4-Mar-21 | 10-Mar-21 | NEGATIVE | 6 | 29-Mar-21 | EGFR NEG WITH OTHER POS | 25 |
| 194 | 4-Mar-21 | 10-Mar-21 | NEGATIVE | 6 | 22-Mar-21 | EGFR NEG WITH OTHER POS | 18 |
| 195 | 5-Mar-21 | 10-Mar-21 | NEGATIVE | 5 | 22-Mar-21 | EGFR NEG WITH OTHER POS | 17 |
| 196 | 5-Mar-21 | 10-Mar-21 | NEGATIVE | 5 | 24-Mar-21 | EGFR NEG WITH OTHER POS | 19 |
| 197 | 8-Mar-21 | 10-Mar-21 | NEGATIVE | 2 | 22-Mar-21 | EGFR NEG WITH OTHER POS | 14 |
| 198 | 8-Mar-21 | 10-Mar-21 | NEGATIVE | 2 | 22-Mar-21 | EGFR NEG WITH OTHER POS | 14 |
| 199 | 8-Mar-21 | 10-Mar-21 | NEGATIVE | 2 | 22-Mar-21 | EGFR NEG WITH OTHER POS | 14 |
| 200 | 8-Mar-21 | 10-Mar-21 | NEGATIVE | 2 | 22-Mar-21 | EGFR NEG WITH OTHER POS | 14 |
| 201 | 8-Mar-21 | 10-Mar-21 | NEGATIVE | 2 | 22-Mar-21 | EGFR NEG WITH OTHER POS | 14 |
| 202 | 9-Mar-21 | 12-Mar-21 | NEGATIVE | 3 | 22-Mar-21 | EGFR NEG WITH OTHER POS | 13 |
| 203 | 9-Mar-21 | 12-Mar-21 | L858R | 3 | 22-Mar-21 | L858R (VAF: 39.5%) | 13 |
| 204 | 9-Mar-21 | 12-Mar-21 | NEGATIVE | 3 | 22-Mar-21 | EGFR NEG WITH OTHER POS | 13 |
| 205 | 10-Mar-21 | 12-Mar-21 | L858R | 2 | 22-Mar-21 | L858R (VAF: 25.8%) | 12 |

| | | | | | | | |
|---|---|---|---|---|---|---|---|
| 206 | 10-Mar-21 | 12-Mar-21 | X19 DELETION | 2 | 24-Mar-21 | X19 DELETION (VAF: 44.4%) | 14 |
| 207 | 12-Mar-21 | 16-Mar-21 | NEGATIVE | 4 | 12-Apr-21 | NO REPORTABLE VARIANTS | 31 |
| 208 | 12-Mar-21 | 16-Mar-21 | L858R | 4 | 8-Apr-21 | L858R (VAF: 14.9%) | 27 |
| 209 | 12-Mar-21 | 16-Mar-21 | NEGATIVE | 4 | 12-Apr-21 | FAIL | 31 |
| 210 | 15-Mar-21 | 17-Mar-21 | L858R | 2 | 29-Mar-21 | L858R (VAF: 16.0%) | 14 |
| 211 | 16-Mar-21 | 18-Mar-21 | NEGATIVE | 2 | 29-Mar-21 | EGFR NEG WITH OTHER POS | 13 |
| 212 | 16-Mar-21 | 18-Mar-21 | NEGATIVE | 2 | 29-Mar-21 | EGFR NEG WITH OTHER POS | 13 |
| 213 | 16-Mar-21 | 18-Mar-21 | NEGATIVE | 2 | 29-Mar-21 | EGFR NEG WITH OTHER POS | 13 |
| 214 | 16-Mar-21 | 18-Mar-21 | NEGATIVE | 2 | 29-Mar-21 | EGFR NEG WITH OTHER POS | 13 |
| 215 | 17-Mar-21 | 19-Mar-21 | NEGATIVE | 2 | 1-Apr-21 | EGFR NEG WITH OTHER POS | 15 |
| 216 | 17-Mar-21 | 19-Mar-21 | NEGATIVE | 2 | 1-Apr-21 | EGFR NEG WITH OTHER POS | 15 |
| 217 | 17-Mar-21 | 19-Mar-21 | NEGATIVE | 2 | 1-Apr-21 | EGFR NEG WITH OTHER POS | 15 |
| 218 | 17-Mar-21 | 19-Mar-21 | NEGATIVE | 2 | 6-Apr-21 | EGFR NEG WITH OTHER POS | 20 |
| 219 | 18-Mar-21 | 22-Mar-21 | X19 DELETION | 4 | 1-Apr-21 | X19 DELETION (VAF: 5.7%) | 14 |
| 220 | 18-Mar-21 | 22-Mar-21 | NEGATIVE | 4 | 1-Apr-21 | NO REPORTABLE VARIANTS | 14 |
| 221 | 18-Mar-21 | 22-Mar-21 | NEGATIVE | 4 | 6-Apr-21 | EGFR NEG WITH OTHER POS | 19 |
| 222 | 18-Mar-21 | 22-Mar-21 | NEGATIVE | 4 | 1-Apr-21 | EGFR NEG WITH OTHER POS | 14 |
| 223 | 19-Mar-21 | 26-Mar-21 | NEGATIVE | 7 | 6-Apr-21 | EGFR NEG WITH OTHER POS | 18 |
| 224 | 19-Mar-21 | 26-Mar-21 | NEGATIVE | 7 | 6-Apr-21 | EGFR NEG WITH OTHER POS | 18 |
| 225 | 19-Mar-21 | 1-Apr-21 | NEGATIVE | 13 | 1-Apr-21 | EGFR NEG WITH OTHER POS | 13 |
| 226 | 23-Mar-21 | 26-Mar-21 | L858R | 3 | 7-Apr-21 | L858R (VAF: 22.8%) | 15 |
| 227 | 24-Mar-21 | 26-Mar-21 | X19 DELETION | 2 | 12-Apr-21 | X19 DELETION (VAF: 55%) | 19 |
| 228 | 25-Mar-21 | 1-Apr-21 | NEGATIVE | 7 | 8-Apr-21 | EGFR NEG WITH OTHER POS | 14 |
| 229 | 25-Mar-21 | 1-Apr-21 | NEGATIVE | 7 | 9-Apr-21 | EGFR NEG WITH OTHER POS | 15 |
| 230 | 26-Mar-21 | 1-Apr-21 | NEGATIVE | 6 | 9-Apr-21 | EGFR NEG WITH OTHER POS | 14 |
| 231 | 26-Mar-21 | 31-Mar-21 | L858R | 5 | 9-Apr-21 | FAIL | 14 |
| 232 | 26-Mar-21 | 1-Apr-21 | NEGATIVE | 6 | 9-Apr-21 | EGFR NEG WITH OTHER POS | 14 |
| 233 | 29-Mar-21 | 7-Apr-21 | NEGATIVE | 9 | 14-Apr-21 | EGFR NEG WITH OTHER POS | 16 |
| 234 | 31-Mar-21 | 7-Apr-21 | NEGATIVE | 7 | 14-Apr-21 | EGFR NEG WITH OTHER POS | 14 |
| 235 | 31-Mar-21 | 7-Apr-21 | NEGATIVE | 7 | 14-Apr-21 | EGFR NEG WITH OTHER POS | 14 |
| 236 | 31-Mar-21 | 7-Apr-21 | NEGATIVE | 7 | 13-Apr-21 | EGFR NEG WITH OTHER POS | 13 |
| 237 | 1-Apr-21 | 1-Apr-21 | L858R | 0 | 12-Apr-21 | FAIL | 11 |
| 238 | 15-Apr-21 | 29-Apr-21 | NEGATIVE | 14 | 28-Apr-21 | EGFR NEG WITH OTHER POS | 13 |
